# Neratinib for HER2-positive breast cancer with an overlooked option

**DOI:** 10.1186/s10020-023-00736-0

**Published:** 2023-10-06

**Authors:** Liting Guo, Weiwei Shao, Chenfei Zhou, Hui Yang, Liu Yang, Qu Cai, Junqing Wang, Yan Shi, Lei Huang, Jun Zhang

**Affiliations:** 1 Department of Oncology, Ruijin Hospital, Shanghai Jiao Tong University School of Medicine, 197 Ruijin Er Road, Shanghai, 200025 China; 2 Department of Pathology, The First People’s Hospital of Yancheng City, Yancheng, China; 3 Department of General Surgery, Ruijin Hospital, Shanghai Jiao Tong University School of Medicine, 197 Ruijin Er Road, Shanghai, 200025 China; 4 Department of General Surgery, Shanghai Seventh People’s Hospital, Shanghai University of Traditional Chinese Medicine, 358 Datong Road, Gaoqiao Town, Shanghai, 200137 China; 5 Medical Center on Aging of Ruijin Hospital, MCARJH, Shanghai Jiaotong University School of Medicine, Shanghai, China

**Keywords:** Neratinib, Breast cancer, HER2, Central nervous system metastasis

## Abstract

Positive human epidermal growth factor receptor 2 (HER2) expression is associated with an increased risk of metastases especially those to the brain in patients with advanced breast cancer (BC). Neratinib as a tyrosine kinase inhibitor can prevent the transduction of HER1, HER2 and HER4 signaling pathways thus playing an anticancer effect. Moreover, neratinib has a certain efficacy to reverse drug resistance in patients with BC with previous HER2 monoclonal antibody or targeted drug resistance. Neratinib, as monotherapy and in combination with other therapies, has been tested in the neoadjuvant, adjuvant, and metastatic settings. Neratinib with high anticancer activity is indicated for the prolonged adjuvant treatment of HER2-positive early BC, or in combination with other drugs including trastuzumab, capecitabine, and paclitaxel for the treatment of advanced HER2-positive BC especially cancers with central nervous system (CNS) metastasis to reduce the risk of BC recurrence. This article reviewed the pharmacological profiles, efficacy, safety, tolerability, and current clinical trials pertaining to neratinib, with a particular focus on the use of neratinib in patients with metastatic breast cancer (MBC) involving the CNS. We further discussed the use of neratinib for HER2-negative and HER2-mutant breast cancers, and mechanisms of resistance to neratinib. The current evidence suggests that neratinib has promising efficacy in patients with BC which is at least non-inferior compared to previous therapeutic regimens. The most common AE was diarrhea, and the incidence, severity and duration of neratinib-related grade 3 diarrhea can be reduced with loperamide. Of note, neratinib has the potential to effectively control and prevent brain metastasis in patients with advanced BC, providing a therapeutic strategy for HER2-positive BC.

## Background

Breast cancer (BC) is the most common cancer among women and the second leading cause of cancer-related death worldwide(Guo et al. 2021; Shah et al. 2018; Shishido et al. 2022). Overexpression of human epidermal growth factor receptor-2 (HER2) occurs in approximately 20–30% of patients with BC and is associated with aggressive behavior, resistance to traditional treatment, and a poor prognosis before the introduction of anti-HER2 agents (Jiang et al. 2018; Martinez-Saez and Prat 2021; Miles and White 2018; Xu et al. 2017). Many of BCs with overexpression of HER2 have a particular tendency to involve the central nervous system (CNS)(Chila et al. 2021; Huang and Xu 2017; Leone and Lin 2019; Schlam and Swain 2021; Xu et al. 2016). Breast cancer brain metastasis (BCBM) is an important cause of morbidity and mortality in patients with MBC (Corti et al. 2022; Leone and Lin 2019; Shah et al. 2018). For the management of BCBM, a multidisciplinary diagnosis and treatment model should be implemented. The purpose of treatment should be to control the metastatic lesions, and to improve the symptoms and quality of life, and to maximize the survival time of patients. BCBM can be treated with surgery, radiotherapy, chemotherapy, and/or supportive care. The general principle of treatment would be to give priority to surgery and/or radiotherapy (SRS and/or WBRT) for CNS lesions under the premise of adequate assessment of the systemic condition, while rationally considering systemic therapy(Freedman et al. 2020). Three major barriers in the CNS limit the effective delivery of drugs: the BBB, the blood-tumor barrier (BTB), and the blood-cerebrospinal fluid barrier (CSF) (Soffietti et al. 2020). The monoclonal antibody trastuzumab has significantly improved outcomes for both early and advanced HER2-positive BC patients(Ban et al. 2020; Baselga et al. 2017; Kourie et al. 2017; Veeraraghavan et al. 2021). Unfortunately, the resistance of trastuzumab will inevitably occur in the majority of cases(Jiang et al. 2018; Martinez-Saez and Prat 2021; Miles and White 2018), and CNS relapses after adjuvant trastuzumab treatment were shown in the HERA trial(Pestalozzi et al. 2013; Schwartz et al. 2019; Sudhan et al. 2019). Trastuzumab is a macromolecular compound, which cannot effectively cross the BBB. However, TKIs are usually small-molecular compounds that can enter CNS. During the last decade many novel drugs such as tyrosine kinase inhibitors (TKIs) and treatment strategies have been developed to overcome the trastuzumab resistance(Huang et al. 2020; Loibl et al. 2021; Martin and Lopez-Tarruella 2016; Oliveira et al. 2020). Therefore, compared with targeted therapy with monoclonal antibody and systemic chemotherapy, small-molecule TKIs have potential advantages in the treatment of CNS metastasis with low toxicity and multiple targeting. When it comes to small-molecule pan-HER-targeted drugs, the most classic usage is in the second and/or third line. Lapatinib is the first reversible small-molecule TKI approved for the treatment of HER2-positive advanced BC(Voigtlaender et al. 2018). It reversibly binds to the ATP-binding site in the tyrosine kinase region of HER1 (EGFR) and HER2 cells, blocking downstream pathways, and thus inhibiting tumor growth(Bilancia et al. 2007; Johnston et al. 2021). In 2007, the US FDA approved the combination of lapatinib and capecitabine for HER2-positive BC that had previously received anthracycline, taxane, and/or trastuzumab(Geyer et al. 2006). In 2010, the US FDA approved lapatinib combined with letrozole for first-line treatment of postmenopausal HR-positive MBC with HER2 overexpression(Lebert and Lilly 2022).

Neratinib (Nerlynx®, HKI-272, Puma Biotechnology Inc., Los Angeles, CA, USA) is an oral, small-molecule, pan-human TKI approved by the US Food and Drug Administration (FDA) in 2017(Dhillon 2019; Iancu et al. 2022; Lopez-Tarruella et al. 2012; Nasrazadani and Brufsky 2020). Neratinib irreversibly binds to the epidermal growth factor receptors (EGFRs) HER1, HER2, and HER4(Dai et al. 2021; Nasrazadani and Brufsky 2020; Paranjpe et al. 2019; Roskoski 2019). In the I-SPY2 neoadjuvant trial, neratinib in combination with trastuzumab resulted in an improvement in the pathologic complete response (pCR) rate in HER2-positive, hormone receptor (HR)-negative BC patients(Park et al. 2016; Stoen et al. 2021). Neratinib has been confirmed to be effective in extended adjuvant therapy following trastuzumab for HER2-positive early BC in the ExteNET trial(Chan et al. 2021; Harbeck 2022; Martin et al. 2017; Singh et al. 2018). In the NEfERT-T trial(Awada et al. 2016), patients with MBC given paclitaxel plus neratinib had lower rates of CNS metastases than those receiving paclitaxel plus trastuzumab, which suggested that neratinib could be effective in BC affecting the CNS. Based on the results of the NALA study(Saura et al. 2020b), the FDA approved neratinib plus capecitabine for patients with advanced HER2-positive BC who have received more than 2 lines of anti-HER2 therapy, with systemic efficacy and intracranial activity.

In this review, we comprehensively searched publications in the PubMed (https://pubmed.ncbi.nlm.nih.gov/) using the following search terms: (Neratinib[title] or Nerlynx[title] or HKI-272[title]) and (breast[title] or mammary[title]) and (cancer[title] or carcinoma[title] or adenocarcinoma[title] or malignancy[title] or neoplasm[title] or tumor[title] or tumour[title]), without language or publication year restrictions. We first described the structure, mechanism, and pharmacological profile of this novel agent-neratinib. We further discussed the phase 1 to 3 clinical trials reporting the efficacy, safety, and tolerability pertaining to the use of neratinib in the treatment of BC, with a focus on the clinical use of neratinib in the management of HER2-positive BC with CNS metastasis. We further discussed the use of neratinib for HER2-negative and HER2-mutant BCs, and mechanisms of resistance to neratinib.

### Structure, mechanism, and pharmacokinetics of neratinib

#### Structure

Neratinib as an anilinoquinoline derivative of pelitinib (EKB-569, Wyeth)(Collins et al. 2019; Feldinger and Kong 2015), is a second-generation TKI against HER1, HER2, and HER4(Aljakouch et al. 2018; Collins et al. 2021; Deeks 2017; Liu et al. 2021). Neratinib derives from 6, 7-disubstituted-4-(arylamino) quinoline-3-carbonitrile(Collins et al. 2019; Tsou et al. 2005), it bonds with *Cys773* of EGFR and *Cys805* of HER2 through a Michael addition reaction(Aljakouch et al. 2018; Feldinger and Kong 2015), and it also experiences extensive conjugation with the Cys residue of glutathione (GSH) by forming a GSH adduct(Feldinger and Kong 2015; Shibata and Chiba 2015). The molecular formula of neratinib is C_30_H_29_ClN_6_O_3_, the compound molecular weight is 673.115, and the chemical formula according to the International Union of Pure and Applied Chemistry (IUPAC) is (E)-N-[4-[3-chloro-4-(pyridin-2-ylmethoxy)anilino]-3-cyano-7-ethoxyquinolin-6-yl]-4-(dimethylamino)but-2-enamide(Shibata and Chiba 2015; Wani et al. 2018).

#### Mechanism and pharmacokinetics

**Fig. 1:**
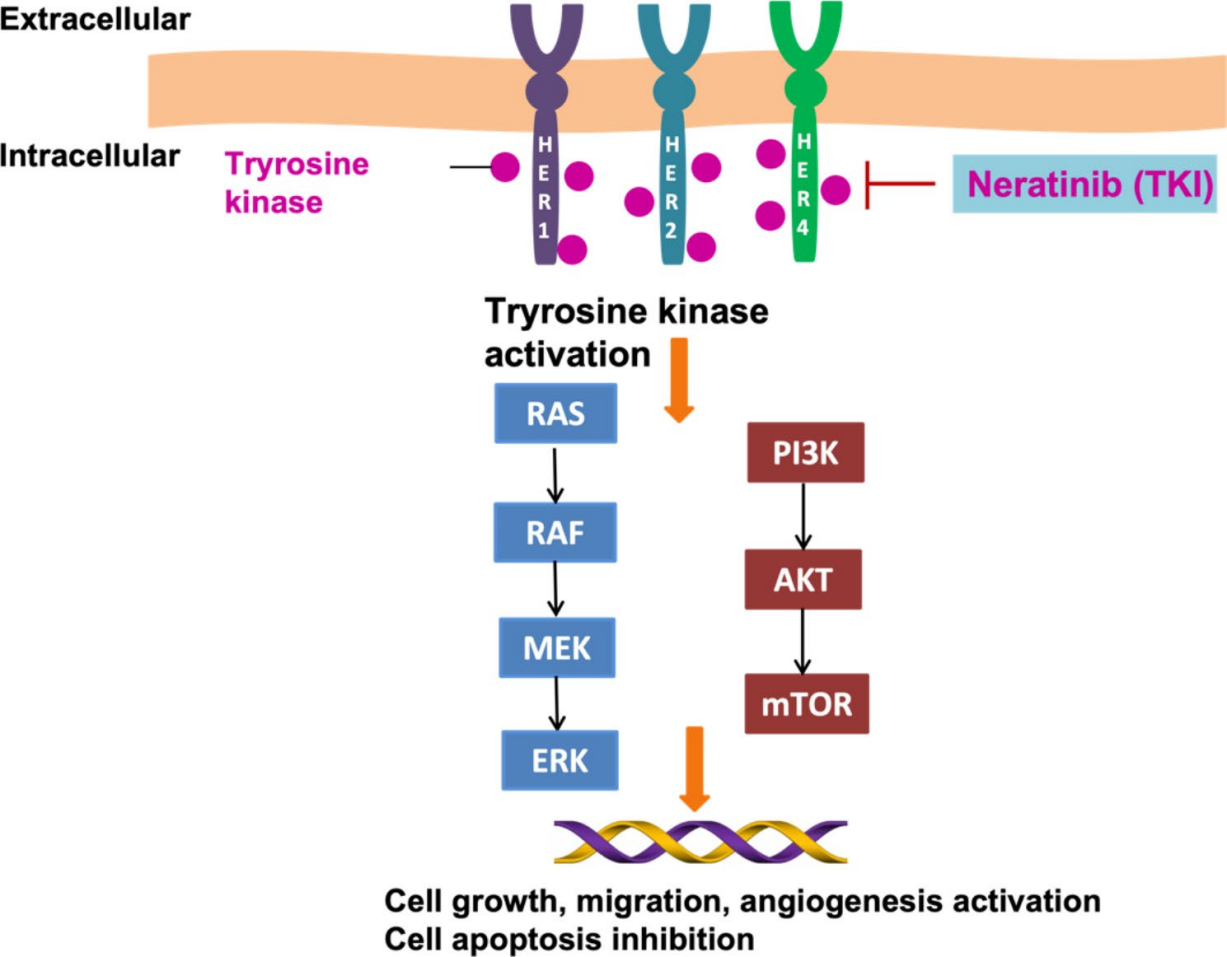
Targeting of the HER1/2/4 receptor with the neratinib blocked breast cancer cell

Neratinib, as an irreversible HER1/2/4 inhibitor, was designed to be a small molecule that could bind to the tyrosine kinase domain and inhibit its interaction with adenosine triphosphate (ATP) in order to prevent receptor phosphorylation(Booth et al. 2018; Chan 2016; Keyvanjah et al. 2017, 2019; Xuhong et al. 2019). Neratinib can reverse multidrug resistance through ATP-binding cassette (ABC) transporters(Nagpal et al. 2019; Zhao et al. 2012). Neratinib inhibits ligand phosphorylated HER2 and EGFR activity, and also inhibits the downstream signaling of the Mitogen-activated protein kinase (MAPK) and AKT pathways(Collins et al. 2019; Deeks 2017; Kourie et al. 2016; Shishido et al. 2022). The main pathways involved included the RAS-RAF-MEK-ERK and PI3K-AKT-mTOR pathways, which could regulate cell proliferation and apoptosis. Neratinib can downregulate the expressions of other RTKs as well as mutant RAS proteins(Dent et al. 2019). It potently inhibits the proliferation of EGFR- and HER2-expressing cell lines, which is associated with G1-to-S-phase cell cycle arrest and apoptosis induction(Keyvanjah et al. 2017; Segovia-Mendoza et al. 2015). Neratinib shows its antitumor activity with also the increase of Protein 27 expression and the decrease of Cyclin D1 expression(Segovia-Mendoza et al. 2015). Neratinib treatment can also activate Heat shock protein 90 (Hsp90) release after HER2 ubiquitination and endocytic degradation(Alkhezayem et al. 2020; Zhang et al. 2016). Neratinib is mainly metabolized by the hepatic CYP3A4 enzyme, and a small proportion is metabolized by Flavin containing Monooxygenase (FMO)(Jerez et al. 2020). It is mainly excreted in feces and has a half-life time of 7 to 17 h. The peak time of the drug is 2 to 8 h after consumption. High-fat meal increases the absorption of neratinib, which is affected by gastric pH(Keyvanjah et al. 2017; Miles and White 2018) (Fig. 1).

### Clinical trials of neratinib use in HER2-positive BC

The efficacy of neratinib for the treatment of tumors has been investigated in several clinical trials, including phase 1, 2, and 3 registered studies (Table 1).

**Table 1:** Summaries of major clinical phase 2/3 trials on TKIs for breast cancer

Study	Registration no.	Cancer type	Country	Trial phase (start year)	No. of subjects	Treatment	Outcome
Awada A, et al.	NCT00706030	Metastatic HER2-positive breast cancer; advanced solid tumors	The US	Phase 1/2 (2013)	92	Neratinib (160 or 240 mg/day, orally) plus vinorelbine (25 mg/m^2^, intravenously; days 1 and 8 of each 21-day cycle)	MTD: 240 mg/day ORR: 41% (without prior lapatinib); 8% (with prior lapatinib).
Chow LW, et al.	NCT00445458	HER2-positive metastatic breast cancer; advanced malignant solid tumors	The US	Phase 1/2 (2013)	110	Neratinib (160 or 240 mg/day orally) plus paclitaxel (80 mg/m^2^ intravenously on days 1, 8, and 15 of each 28-day cycle)	MTD: 240 mg/day ORR: 73% Median PFS: 57 weeks
Saura C, et al.	NCT00741260	HER2-positive metastatic breast cancer	The US	Phase 1/2 (2014)	105	Neratinib (160, 200, or 240 mg/day orally) plus capecitabine (1500 or 2000 mg/m^2^ orally per day on days 1 to 14 of a 21-day cycle)	MTD: 240 mg/day ORR: 64% (without previous lapatinib); 57% (with previous lapatinib) Median PFS: 40.3 weeks (without previous lapatinib); 35.9 weeks (with previous lapatinib)
Burstein HJ, et al.	NCT00300781	Advanced HER2-positive breast cancer	Multinational	Phase 2 (2010)	136	Neratinib 240 mg/day orally	ORR: 24% (with prior trastuzumab); 56% (without prior trastuzumab) Median PFS: 22.3 weeks (with prior trastuzumab); 39.6 weeks (without prior trastuzumab)
Martin M, et al.	NCT00777101	Advanced HER2-positive breast cancer	Multinational	Phase 2 (2013)	233	Neratinib 240 mg/day orally monotherapy vs. lapatinib (1250 mg/day) plus capecitabine (2000 mg/m^2^/day on days 1–14 of each 21-day cycle)	Median OS: 19.7 vs. 23.6 months Median PFS: 4.5 vs. 6.8 months ORR: 29% vs. 41%
Freedman RA, et al.	NCT01494662 (TBCRC 022)	HER2-Positive breast cancer and brain metastases	Multinational	Phase 2 (2016)	40	Neratinib 240 mg/day orally	ORR: 8% Median OS: 8.7 months Median PFS:1.9 months
Park JW, et al.	NCT01042379	Early breast cancer	The US	Phase 2 (2016)	193	Neratinib 240 mg/day orally vs. trastuzumab (loading dose of 4 mg/kg intravenously for the first cycle, followed by maintenance dose of 2 mg/kg for cycles 2–12)	pCR rate: 56% vs. 33%
Awada A, et al.	NCT00915018 (NEfERT-T)	Metastatic HER2-positive breast cancer	Multinational	Phase 2 (2009)	479	Neratinib (240 mg/day orally) or trastuzumab (4 mg/kg then 2 mg/kg intravenously weekly), each combined with paclitaxel (80 mg/m^2^ intravenously on days 1, 8, and 15 every 28 days)	Median PFS: 12.9 vs. 12.9 months ORR: 74.8% vs. 77.6% CBR: 88.4% vs.85.2% Median DOR: 13.4 vs. 12.9 months Incidence of CNS recurrence: 8.3% vs. 17.3%
Jacobs SA, et al.	NCT01008150 (NSABP FB-7)	Locally advanced HER2-positive breast cancer	Multinational	Phase 2 (2011)	126	Arm 1: Paclitaxel (80 mg/m^2^ intravenously on days 1, 8, and 15 of a 28-day cycle) with trastuzumab (4 mg/kg intravenously as loading dose, and then 2 mg/kg weekly for a total of 16 doses) Arm 2: Paclitaxel (80 mg/m^2^ intravenously on days 1, 8, and 15 of a 28-day cycle), and neratinib (240 mg/day orally) Arm 3: Trastuzumab and paclitaxel were intravenously given as in Arm 1, and neratinib (200 mg/day orally)	pCR rate: 38% vs. 33% vs. 50%
Chan A, et al.	NCT00878709 (ExteNET)	Early stage HER2-positive breast cancer	Multinational	Phase 3(2009)	2840	Neratinib (240 mg once daily orally) vs. placebo	2-year iDFS: 93.9% vs. 91.6% 5-year iDFS: 90.2% vs. 87.7%
Saura C, et al.	NCT01808573 (NALA)	Metastatic HER2-positive breast cancer	Multinational	Phase 3(2013)	621	Neratinib (240 mg once daily orally) plus capecitabine (750 mg/m^2^ orally twice a day for 14 days of a 21-day cycle) with loperamide prophylaxis, or lapatinib (1250 mg once daily orally) plus capecitabine (1000 mg/m^2^ orally twice a day for 14 days of a 21-day cycle)	Mean PFS: 8.8 vs. 6.6 months Median PFS: 5.6 vs. 5.5 months Mean OS: 24.0 vs. 22.2 months Intervention for CNS disease: Cumulative incidence: 22.8% vs. 29.2% ORR: 32.8% vs. 26.7% Median DOR: 8.5 vs. 5.6 months
Geyer CE, et al.	NCT00078572	HER2-positive advanced breast cancer	The US	Phase 3(2004)	324	Combination therapy (lapatinib at a dose of 1250 mg per day continuously plus capecitabine at a dose of 2000 mg per square meter of body-surface area on days 1 through 14 of a 21-day cycle) or monotherapy (capecitabine alone at a dose of 2500 mg per square meter on days 1 through 14 of a 21-day cycle)	Median PFS: 8.4 vs. 4.4 months
Goss PE, et al.	NCT00374322	HER2-positive early breast cancer	Multinational	Phase 3(2006)	3161	Daily lapatinib (1500 mg) or daily placebo	Median DFS: 47.4 vs. 48.3 months
Baselga J, et al.	NCT00553358 (NeoALTTO)	HER2-positive early breast cancer	Multinational	Phase 3(2008)	455	Oral lapatinib (1500 mg), intravenous trastuzumab (loading dose 4 mg/kg, subsequent doses 2 mg/kg), or lapatinib (1000 mg) plus trastuzumab for the first 6 weeks; weekly paclitaxel (80 mg/m2) was then added to the regimen for a further 12 weeks	pCR rate: 51.3% vs. 29.5%
Ma F, et al.	NCT02422199	HER2-positive metastatic breast cancer	China	Phase 2 (2015)	128	400 mg pyrotinib or lapatinib 1250 mg orally once per day for 21-day cycles in combination with capecitabine (1000 mg/m^2^ orally twice per day on days 1 to 14)	ORR: 78.5% vs. 57.1% Median PFS: 18.1 vs. 7.0 months
Yan M, et al.	NCT02973737 (PHENIX)	HER2-positive metastatic breast cancer	China	Phase 3 (2016)	279	Pyrotinib or placebo (400 mg, qd) plus capecitabine (1,000 mg/m^2^, bid on days 1–14) for 21-day cycles	Median PFS: 11.1 vs. 4.1 months
Xu B, et al.	NCT03080805 (PHOEBE)	HER2-positive metastatic breast cancer	China	Phase 3 (2017)	267	Pyrotinib 400 mg or lapatinib 1250 mg once daily plus oral capecitabine 1000 mg/m^2^ twice daily on days 1–14 of each 21-day cycle	Median PFS: 12.5 vs. 6.8 months
Kim et al.	NCT02418689 (NOV120101-203 trial)	HER2-positive metastatic breast cancer	South Korea	Phase 2 (2015)	106	12 mg poziotinib once daily on a 14-day on/7-day off schedule	Median PFS: 4.04 months
Curigliano G, et al.	NCT02614794	HER2-positive metastatic breast cancer	Multinational	Phase 3 (2015)	612	Tucatinib (oral; 300 mg twice daily), trastuzumab (6 mg/kg every 3 weeks), plus capecitabine (1000 mg/m^2^ twice daily on days 1–14 every 21 days)	Median OS: 24.7 vs. 19.2 months 2-year OS rate: 51% vs. 40% Median PFS: 7.6 vs. 4.9 months 1-year PFS rate: 29% vs. 14%

MTD, maximum tolerated dose; PR, partial response; SD, stable disease; ORR, objective response rate; OS, overall survival; CR, complete response; TTP, time to disease progression; DFS, disease-free survival; PFS, progression-free survival; pCR, pathologic complete response; iDFS, invasive disease-free survival; CNS, central nervous system; DOR, duration of response

#### Phase 1

The first in-human phase 1 neratinib trial, was designed as an open-label study to discuss the dose-limiting toxicity (DLT), maximum tolerated dose (MTD), and pharmacokinetic profile of neratinib(Wong et al. 2009). The trial consisted of a total of 72 patients with HER2- or EGFR-positive solid tumors including BC (40%), non-small-cell lung cancer (NSCLC; 21%), ovarian cancer (8%), colorectal cancer (6%), glioblastoma (6%), renal cancer (4%), pancreatic cancer (3%), and other cancers (13%)(Chila et al. 2021; Wong et al. 2009). The patients initially received single oral doses of neratinib of 40, 80, 120, 180, 240, 320, 400, and 500 mg daily taken with food, followed by 1-week observation to assess the single-dose pharmacokinetic profiles and adverse events (AEs). Neratinib was well tolerated, with the most common AE being diarrhea (88%), grade 3 or higher neratinib-related adverse events (AEs) occurred in 39% of all patients, and the MTD was determined to be 320 mg; 25 patients with BC were evaluated for treatment efficacy, with partial response (PR) observed in 8 (32%) cases, and stable disease (SD) in 1 (4%) case(Wong et al. 2009).

In another multicenter, open-label, dose-escalation phase 1 study of neratinib in Japanese patients with advanced solid tumors, 21 patients (including 3 BC patients) were enrolled, the MTD of neratinib was also determined to be 320 mg(Ito et al. 2012). Two patients with BC had PR. Grade 3 neratinib-related AEs in two or more patients were diarrhea and anorexia. The safety, efficacy, and pharmacokinetic profiles of neratinib were consistent with those reported for non-Japanese patients.

Another open-label phase 1 dose-escalation trial by the NSABP Foundation Research Program recruited 21 patients to further determine the MTD, safety, and efficacy of neratinib (120 to 240 mg/day) with trastuzumab and paclitaxel in HER2-positive MBC previously treated with anti-HER agent(s) and a taxane(Jankowitz et al. 2013). Objective responses occurred in 8 patients (38%), and complete response (CR) + PR + SD for ≥ 24 weeks in 11 patients (52%); the median time-to-tumor progression (TTP) was 3.7 months. In terms of safety, gastrointestinal toxicity such as diarrhea remained the most common adverse event associated with neratinib use, grade 3 diarrhea occurred in 8 patients (38%), no patients experienced grade 4 diarrhea (Deeks 2017; Jankowitz et al. 2013).

A subsequent multicenter, open-label, dose-escalation, phase 1b study (NSABP Foundation Trial FB-10) evaluated trastuzumab-emtansine (T-DM1) plus neratinib in women with HER2-positive MBC; 5 institutions enrolled 27 patients who progressed on trastuzumab, pertuzumab, and a taxane, and they were treated with T-DM1 (3.6 mg/kg intravenously every 3 weeks) and dose-escalating neratinib (120, 160, 200, or 240 mg/day orally) in the trial(Abraham et al. 2019). 63% evaluable patients at all neratinib doses had an objective response, and 7% had CR. The study also suggested that the mechanism of HER2 antibody resistance may lie in the internalization of p185 HER2 and the high expression of p95 HER2(Abraham et al. 2019). Diarrhea was the most common AE, with all grades occurring in 23 patients (93%) and grade 3 in 22%. Diarrhea developed early during treatment could be easily controlled and well managed by temporarily discontinuing or reducing drug dose and/or using intensive antidiarrheal medications as prophylaxis.

The most common AE of neratinib was gastrointestinal toxicity such as diarrhea; in patients with BC, the response rate of neratinib was ≥ 32%, and was higher when used together with other anti-HER2 agents (e.g., 63% when with T-DM1). These phase 1 data lay the foundation for further phase 2 studies to better determine the efficacy and safety of the neratinib-based regimens. The MTD of neratinib in phase 1 dose is 320 mg/day, but additional clinical experience indicated unacceptable rates of diarrhea. As monotherapy, the dose of 240 mg/day was used for the Phase 2 studies.

#### Phase 1/2

A phase 1/2 trial conducted by Awada A et al. evaluated the safety and efficacy of neratinib and vinorelbine in patients with solid tumors including HER2-positive MBC (12 patients were enrolled in phase 1, and 79 patients in phase 2)(Awada et al. 2013). In phase 1 with 12 patients, neratinib (240 mg) plus vinorelbine (25 mg/m^2^) was established as the MTD; in phase 2 with 79 patients, the objective response rate (ORR) was 41% (without prior lapatinib) and 8% (with prior lapatinib)(Awada et al. 2013). A total of 26 (29%) patients experienced grade 3 diarrhea, 3 (3%) patients discontinued treatment due to diarrhea.

In another phase 1/2, open-label, two-part study, the MTD of oral neratinib (240 mg once daily) plus intravenous paclitaxel (80 mg/m^2^ on Days 1, 8, and 15 of each 28-day cycle) was determined. The trial tested neratinib and paclitaxel in a total of 102 patients in Part 2. The median overall treatment duration was 47.9 weeks. Among the 99 evaluable patients, the ORR was 73%, 7 (7%) patients had CR, and 9 (9%) patients achieved SD for at least 24 weeks. The median progression-free survival (PFS) was 57.0 weeks(Chow et al. 2013). 29% patients experienced grade 3 diarrhea, there were no grade 4 events of diarrhea reported.

Another multinational, open-label, phase 1/2 trial also included two parts. Part 1 (3 + 3 dose-escalation study) was to evaluate the MTD of neratinib (once per day) combined with capecitabine (twice per day on Days 1 to 14 of a 21-day cycle) in 33 patients with advanced solid tumors; Part 2 recruited 72 patients with trastuzumab-pretreated HER2-positive MBC, and the safety and efficacy of neratinib plus capecitabine were evaluated. The MTD was 240 mg per day for neratinib, and 1500 mg/m^2^ per day for capecitabine, respectively. The ORR was 64% (39/61 with no prior lapatinib) and 57% (4/7 previously treated with lapatinib), and the median PFS was 40.3 and 35.9 weeks, respectively(Saura et al. 2014). Together, when used with vinorelbine, paclitaxel, or capecitabine for BC, the response rate of neratinib could be as high as 73% and varied according to previous targeted therapy, with a MTD of 240 mg and longest median PFS of up to 57.0 weeks. The incidence of grade 3/4 diarrhea was 23%, as expected from the combination of neratinib plus capecitabine associated with GI effects.

#### Phase 2

Based on the above phase 1 or 1/2 data, an open-label, multicenter, phase 2 trial was launched to assess the clinical activity of neratinib in two cohorts of patients with advanced HER2-positive BC (66 with prior trastuzumab treatment, and 70 with no prior trastuzumab treatment)(Burstein et al. 2010). A total of 136 patients with Stage IIIB, IIIC, or IV BC were treated with neratinib 240 mg once daily according to the results of phase 1 trials. The results showed that the median PFS time for 63 patients with prior trastuzumab treatment and 63 with no prior trastuzumab treatment was 22.3 and 39.6 weeks, respectively; the 16-week PFS rates were 59% and 78%, and the ORRs 24% and 56%, respectively(Burstein et al. 2010). Grade 3–4 diarrhea were occurring in 30% of patients with prior trastuzumab treatment and in 13% of patients with no prior trastuzumab treatment.

In another open-label, randomized, multicenter phase 2 trial, 233 patients were randomly assigned to receive neratinib 240 mg/d (117 patients) or lapatinib 1250 mg/d plus capecitabine 2000 mg/m^2^ per day on Days 1–14 of each 21-day cycle (116 patients). The primary aim was to illustrate the non-inferiority of neratinib treatment regarding PFS. Among patients with locally advanced or metastatic HER2-positive BC not amenable to curative surgery and/or radiation therapy, the median PFS was 4.5 months for the neratinib group and 6.8 months for the lapatinib plus capecitabine group (*P* = 0.231), and the OS was 19.7 and 23.6 months (*P* = 0.280), respectively(Martin et al. 2013). The ORR was 29% in the neratinib group versus 41% in the lapatinib plus capecitabine group (*P* = 0.067), and the clinical benefit rates of the two groups were 44% and 64% (*P* = 0.003), respectively(Martin et al. 2013). These findings suggested that neratinib monotherapy was inferior to the combination therapy modality with lapatinib plus capecitabine. Grade 3 diarrhea occurred in 28% patients with neratinib group, 10% patients with lapatinib plus capecitabine group.

The expansion cohort of the multicenter, phase 2 neoadjuvant I-SPY2 trial studied the effects of multiple new agents added to standard chemotherapy on the rates of pathological complete response. 115 patients with high-risk clinical stage II/III HER2-positive HR-negative BC who received neratinib in addition to chemotherapy and 78 concurrently randomized controls who received weekly paclitaxel alone were enrolled, and the pCR rate was 56% for the neratinib group and 33% for the control group. Grade 3–4 diarrhea was noted in 38% of patients in the neratinib arm (Park et al. 2016; Wulfkuhle et al. 2018).

The open-label randomized controlled phase 2 NEfERT-T trial compared neratinib (240 mg/d orally) plus paclitaxel (80 mg/m^2^ on days 1, 8, and 15 every 28 days) with trastuzumab (4 mg/kg followed by 2 mg/kg weekly) plus paclitaxel in 479 previously-treated patients (242 receiving neratinib-paclitaxel and 237 trastuzumab-paclitaxel) with recurrent and/or metastatic HER2-positive BC; women with asymptomatic CNS involvement were eligible, and the randomization was stratified by prior trastuzumab and lapatinib exposure, HR status, and region. The median PFS time of the neratinib-paclitaxel group and the trastuzumab-paclitaxel group was both 12.9 months (*P* = 0.89)(Awada et al. 2016). The ORRs were 74.8% and 77.6% (*P* = 0.52), respectively, and the clinical benefit rates (CBRs) 88.4% and 85.2% (*P* = 0.24), respectively. The median duration of response (DOR) was also similar between both groups (*P* = 0.92). However, notably, the incidence of CNS recurrence in the neratinib-paclitaxel group (10.1%) was lower than that in the trastuzumab-paclitaxel group (20.2%) (*P* = 0.002)(Awada et al. 2016), and the time to CNS metastasis was also delayed in the neratinib-paclitaxel group (16.3% in the neratinib-paclitaxel group vs. 31.2% in the trastuzumab-paclitaxel group, *P* = 0.004)(Awada et al. 2016), which suggested that the neratinib-paclitaxel regimen could have delayed the metastatic CNS disease. Grade 3 diarrhea occurred in 30.4% of patients in the neratinib-paclitaxel group and 3.8% of patients in the trastuzumab-paclitaxel group, and no grade 4 diarrhea was observed.

Freedman RA et al.(Freedman et al. 2016) conducted a multicenter, open-label phase 2 trial, the Translational Breast Cancer Research Consortium (TBCRC) 022 trial, where 40 patients with HER2-positive BC who had brain metastasis (≥ 1 cm in the longest dimension) received neratinib 240 mg once daily as monotherapy; these patients had progressed over previous CNS-directed therapy, such as whole brain radiotherapy (WBRT), stereotactic radiosurgery (SRS), surgery, or any combination. Composite CNS objective response required all of the following: ≥50% reduction in the sum of target CNS lesion volume, no progression of non-target lesions, no new lesions, no escalating steroids, no progressive neurologic symptoms or signs, and no non-CNS progression. The ORR of the CNS lesion as the primary endpoint was 8%, and only three cases achieved a PR; the median PFS was 1.9 months and the median OS 8.7 months(Freedman et al. 2016). In cohort 3 A of the phase 2 TBCRC 022 trial, patients with BC and CNS metastasis were specifically recruited, and the main purpose was to explore the ORR of neratinib combined with capecitabine in patients with BCBM; the composite ORR was 49% in cohort 3 A (n = 37), and 33% in cohort 3B (n = 12)(Bradley 2019; Freedman et al. 2019; Freedman et al. 2016). Diarrhea was the most common grade 3 toxicity (29% in cohorts 3 A and 3B).

In another phase 2 NSABP FB-7 neoadjuvant trial by Jacobs SA et al., 126 patients with locally advanced HER2-positive BC were randomly divided into 3 groups (1:1:1): Control group with trastuzumab (4 mg/kg loading dose, followed by 2 mg/kg weekly) + paclitaxel (60 mg/m^2^ weekly) (T + P), experimental group with neratinib (240 mg) + paclitaxel (N + P), and combination group (trastuzumab + neratinib + paclitaxel; T + N + P). All the treatment was followed by standard doxorubicin (60 mg/m^2^) plus cyclophosphamide (600 mg/m^2^) (AC)(Jacobs et al. 2019; 2020). The pCR rate as the primary endpoint was 50.0% in the combination group, 38.1% in the trastuzumab-paclitaxel group, and 33.3% in the neratinib-paclitaxel group(Jacobs et al. 2019). Together, the median PFS for neratinib was 4.5 to 12.9 months, and the ORR was 24–75%, which varied according to previous targeted therapy and combined chemotherapy. Together with chemotherapy, the pCR rate could be as high as 56%. Grade 3 diarrhea reported in 31% of patients.

The randomized phase 2 studies (I-SPY2 and NSABP FB-7) supported the efficiency of neratinib with standard chemotherapy in the neoadjuvant setting. Notably, neratinib appeared CNS metastasis-protective.

#### Phase 3

Based on the above appealing phase 2 data, a multicenter, randomized, double-blind, placebo-controlled, phase 3 trial (the ExteNET trial) was conducted, where 2840 patients with previously trastuzumab-treated stage II-III HER2-positive BC were randomly assigned (1:1) to receive neratinib (n = 1420) and placebo (n = 1420)(Chan et al. 2016; Chia et al. 2019). The study was to investigate the efficacy and safety of 12-month neratinib use after trastuzumab-based adjuvant therapy in patients with early-stage HER2-positive BC. The 2-year invasive disease-free survival (iDFS) rate was 93.9% in the neratinib group and 91.6% in the placebo group (*P* = 0.0091)(Chan et al. 2016). The 5-year iDFS rate was 90.2% in the neratinib group and 87.7% in the placebo group (*P* = 0.0083)(Martin et al. 2017; Unni et al. 2018). 561 (40%) patients had grade 3 diarrhea, and one patient had grade 4 diarrhea. The AEs were consistent with the previous report(Chan et al. 2021). The cumulative incidence of first CNS recurrences at 5 years was 0.7% with neratinib and 2.1% with placebo. At 5 years, 98.4% of patients in the neratinib group and 95.7% of patients in the placebo group were alive and did not report a CNS recurrence (hazard ratio for CNS-DFS 0.41). Together, neratinib prevented CNS-associated events in patients with BC. In the ExteNET study, extended consolidation therapy with neratinib was continued for early-stage HER2-positive BC with disease control following trastuzumab plus chemotherapy. The ExteNET study showed that neratinib given within 1 year of trastuzumab significantly improved iDFS in patients with HER2-positive BC, and that HR-positive patients who started neratinib within one year of trastuzumab could benefit from the treatment. Based on the results of the ExteNET trial, neratinib was approved by the US FDA in July 2017 as monotherapy for extended adjuvant treatment of HER2-positive early-stage BC.

Another the phase 3 NALA trial (NCT01808573) is a global multicenter, open-label, randomized clinical trial(Mo et al. 2021; Saura et al. 2020b). The NALA study included a total of 621 patients with HER2-positive MBC who had received at least 2 prior targeted therapies to assess the efficacy and safety of neratinib plus capecitabine (N + C) versus lapatinib plus capecitabine (L + C). Eligible patients with HER2-positive MBC aged ≥ 18 years, with ECOG score ≤ 1, and having received ≥ 2 types of HER2-targeted therapies. 101 (16.3%) patients with brain metastases (BMs) were only eligible if they had asymptomatic, stable BMs. The study was divided into two groups based on treatment, the N + C group and the L + C group, and all patients were randomized at the ratio of 1:1. A total of 307 patients in the N + C group took neratinib 240 mg orally once a day continuously in the 21-day cycles, and a total of 314 patients in the L + C group took lapatinib 1250 mg orally once a day continuously. Both groups were administered capecitabine orally twice a day at a dose of 750 mg/m^2^ at the same time on days 1–14 of the 21-day cycles. A 21-day cycle of treatment was performed until the disease progressed or intolerable toxicity and/or side effects occurred. The primary endpoints were PFS and OS, and secondary endpoints ORR, DOR, CBR, time to intervention for CNS disease, and safety. The results showed that the N + C regimen was superior to the L + C regimen (Table 2). The N + C group had better performance than the L + C group with numerically prolonged PFS and OS; the PFS time was statistically different between the 2 groups (up to 2.2 months), but a statistical difference was not reached regarding OS time. In the Chinese subgroup of the phase 3 NALA study which aimed to compare the efficacy of N + C versus L + C in patients with previously treated HER2-positive MBC, there was a 62% relative reduction in the risk of progression or death, and the OS benefit (23.8 months in the N + C group vs. 15.4 months in the L + C group) was statistically significant. Regarding the control of BM, the time to symptomatic CNS metastasis was delayed in the N + C group compared with the L + C group (overall incidence, 22.8% vs. 29.2%, *P* = 0.043). Grade 3 diarrhea occurred in 74 patients (24.4%) with neratinib and 39 patients (12.5%) with lapatinib. Based on the results of the NALA study, the FDA approved neratinib plus capecitabine for patients with advanced HER2-positive MBC who have received more than 2 lines of anti-HER2 therapy(Saura et al. 2020b).

**Table 2:** Summary of efficacy endpoints of the NALA trial (neratinib), HER2Climb trial (tucatinib) and PHENIX trial (pyrotinib)

**Endpoint (NALA trial)**	**Neratinib + capecitabine (N + C)**	**Lapatinib + capecitabine (L + C)**	***P***
Mean PFS	8.8 months	6.6 months	0.0059
Median PFS	5.6 months	5.5 months	
6-month PFS rate	47.2%	37.8%	
12-month PFS rate	28.8%	14.8%	
18-month PFS rate	16.3%	7.4%	
Mean OS	24.0 months	22.2 months	0.2086
Median OS	21.0 months	18.7 months	
Cumulative incidence of intervention for CNS disease	22.8%	29.2%	0.0430
ORR	32.8%	26.7%	0.1201
DOR	8.5 months	5.6 months	0.0004
**Endpoint (HER2CLIMB trial)**	**Tucatinib combination**	**Placebo combination**	***P***
Median PFS	7.6 months	4.9 months	< 0.00001
1-year PFS rate	29%	14%	
Median OS	24.7 months	19.2 months	0.004
2-year OS rate	51%	40%	
Median CNS-PFS	9.9 months	4.6 months	
Median CNS-OS	21.6 months	12.5 months	
Confirmed ORR-IC	47.3%	20.0%	
median DOR-IC	8.6%	3.0%	
**Endpoint (PHENIX trial)**	**Pyrotinib + capecitabine**	**Placebo + capecitabine**	***P***
Median PFS	11.1 months	4.1 months	< 0.001
Median CNS-PFS	6.9 months	4.2 months	0.011
ORR	68.6%	16.0%	< 0.001
DOR	12.2 months	4.2 months	< 0.001
DCR	91.9%	64.9%	< 0.001
CBR	76.8%	22.3%	< 0.001

PFS, progression-free survival; OS, overall survival; CNS, central nervous system; ORR, objective response rate; DOR, duration of response; DOR-IC, duration of intracranial response; ORR-IC, intracranial objective response rate, Tucatinib combination: tucatinib, trastuzumab, and capecitabine; Placebo combination: placebo, trastuzumab, and capecitabine; CBR, clinical benefit rate

Together, neratinib is a promising regimen for HER2-positive BC in both the neoadjuvant and adjuvant settings, as well as for MBC, and is CNS metastasis-protective. The efficacy differed according to previous anti-HER2 therapy and combined chemotherapy. The treatment modality using neratinib in the neoadjuvant and adjuvant settings for HER2-positive BC has been evolving Anti-HER2 targeted therapy showed significant survival benefits for both early and advanced BC.

### Neratinib efficacy in HER2-positive BC with CNS metastasis

BC is prone to metastasis to the brain(Duchnowska et al. 2018; Lin et al. 2020). The incidence of CNS metastasis varies by BC subtype and stage. HER2 overexpression is related to an increased risk of CNS metastasis, and the proportion of cases with CNS metastasis in HER2-positive BC patients ranges from 30–55%(Aleanizy et al. 2020; Zimmer et al. 2020). Overexpression or abnormal amplification of HER2 makes BC cells more aggressive and easier to metastasize to the CNS(Hurvitz et al. 2021). Although anti-HER2 targeted therapy prolongs the survival of BC patients, and improves the control rate of extracranial lesions, the existence of the blood-brain barrier greatly weakens the killing effect of most systemic drugs on brain tumors. Once HER2-positive BC develops brain metastasis, the effective management of such patients has become a major clinical challenge(Freedman et al. 2019; Soffietti et al. 2020; Stavrou et al. 2021).

Targeted therapy has always occupied a core position in the field of HER2-positive BC treatment. There are some regimens targeting HER-positive BC, from trastuzumab alone to trastuzumab in combination with pertuzumab, and then to oral small-molecule drugs like lapatinib. In addition, T-DM1 is approved for use in patients with HER2-positive MBC who previously received treatment with trastuzumab and a taxane. In the KATHERINE study, the CNS was more often the site of first recurrence in the T-DM1 arm (5.9% versus 4.3%), T-DM1 does not decrease the risk of brain relapses (Mamounas et al. 2021). With the advent of trastuzumab and the debut of the upgraded antibody-drug conjugate T-DM1(Paracha et al. 2020), targeted therapy ushered another highlight moment(Cesca et al. 2020; Modi et al. 2020; Prove and Dirix 2016).

Lapatinib is a reversible dual-target HER2-directed TKI(Jacobs et al. 2019), and could to some extent control brain metastasis. However, for patients with refractory HER2-positive MBC who have previously received at least 2 targeted therapies, lapatinib-based treatment options appear to be stretched. Notably, neratinib, as a new HER2-targeted TKI, has the characteristics of irreversibly binding more targets. Neratinib has been shown to be effective against BM in HER2-positive BC patients in several studies, especially a phase 2 TBCRC 022 trial(Freedman et al. 2016), which indicates that neratinib combined with capecitabine is effective against BCBM. The ORR in the patients was 49%, and patients who were resistant to lapatinib could still achieve an ORR of 33%. After the above first phase 2 clinical study on neratinib for BCBM, the most pivotal phase 3 NALA trial study was done and described in detail in the Phase 3 above. Based on the results of NALA trial, in February 2020, neratinib was approved by the US FDA with the expansion of the scope of application to be in combination with capecitabine for the treatment of adult patients with advanced or metastatic HER2-positive BC who have received two or more prior anti-HER2 regimens.

Notably, the current evidence on the efficacy and safety of neratinib for the treatment of HER2-positive BC mainly derives from Western patients. Racial differences in tumor biology of BC may lead to different outcomes, and treatment patterns vary by region; it is necessary to analyze the efficacy and safety of neratinib in Asian patients with advanced BC(Iwata et al. 2019; Xu et al. 2019). Therefore, a subgroup analysis of this study based on Asian patients with HER2-positive advanced BC who failed ≥ 2 HER2-targeted therapies was performed, revealing the same efficacy characteristics in Asian patients as in the entire study population; thus, Asian patients could also significantly benefit from neratinib plus capecitabine, with no new security issues having been observed(Dai et al. 2021).

Together, when used with capecitabine, the ORR of neratinib in patients with BCBM was 8–49%, which varied according to treatment history and other HER2-targeting drug resistance, and which was superior to lapatinib plus capecitabine. Neratinib was also brain metastasis-preventative, and significantly reduced brain metastasis rate and prolonged time to brain metastasis.

### HER2-positive and HR-positive advanced BC

According to molecular typing, BC can be divided into three types: HER-2 positive, triple negative and HR positive breast cancer. Among them, HER-2 positive BC is a type of HER-2 overexpressed BC, which accounts for about 20% of all BC, and about 50% of them are HER-2 positive and HR positive patients. In endocrine therapy resistant BC, HER-2 mutation frequency was increasing and PI3K/Akt/mTOR signaling pathway is over activated, at this time, neratinib was given to inhibit the HER-2 function of mutation, it can not only effectively control the tumor, but also restore the efficacy of anti-hormone therapy(Croessmann et al. 2019). In HER-2 positive and HR positive tumor xenotransplantation mice, after the treatment of paclitaxel combined with trastuzumab ± pertuzumab, the mice treated with single drug fulvestrant relapsed rapidly, while the mice treated with neratinib plus fulvestrant maintained a longer time of tumor remission(Sudhan et al. 2019). Estrogen receptors (ER) blockade induced by endocrine therapy can lead to reactivation of the HER receptor tyrosine kinase pathway, thus prolonging HER pathway blockade improves the prognosis of HER-2 positive and HR-positive BC(Sudhan et al. 2019).In addition, neratinib can overcome the drug resistance of trastuzumab and traditional chemotherapy drugs(Gamez-Chiachio et al. 2022). In trastuzumab resistant cell lines and mouse models, the anti-tumor effect of trastuzumab combined with neratinib is more obvious than trastuzumab or neratinib alone. In addition, in the exploration of intensive adjuvant chemotherapy strategy, neratinib can improve the iDFS of HER-2 positive early BC patients, reduce the risk of recurrence, and benefit more significantly in HER-2 positive and HR positive BC patients(Chan et al. 2021). Although there is a lack of data related to neratinib for advanced HER-2 positive and HR positive BC, however, targeted therapy in combination with endocrine therapy has shown initial success in the treatment of this population.

### Safety and adverse events associated with neratinib

The majority of AEs associated with neratinib were generally mild to severe in severity (grade 1–3), and grade 4 events were rare(Chan et al. 2016). The most common AEs of neratinib (240 mg/day) are diarrhea, nausea, fatigue, vomiting, abdominal pain, headache, rash, decreased appetite, muscle spasms, dizziness, and arthralgia(Chan et al. 2016; Mortimer et al. 2019). In the NALA trial(Saura et al. 2020b), the AEs occurring in the neratinib plus capecitabine (N + C) group and the lapatinib plus capecitabine (L + C) group were similar, and included diarrhea (83.0%), nausea (53.1%), palmar-plantar erythrodysesthesia (PPE) syndrome (45.9%), vomiting (45.5%), decreased appetite (35.3%), constipation (31.0%), stomatitis (20.5%), weight decrease (19.8%), rash (9.9%), anemia (14.9%), dizziness (14.2%), cough (12.2%), abdominal pain (11.9%), asthenia (11.9%), hypokalemia (11.6%), paronychia (11.6%), pyrexia (10.9%), and headache (10.6%). Grade 3 diarrhea occurred in 74 patients (24.4%) with neratinib. The N + C group had fewer discontinuations due to AEs than the L + C group (10.9% vs. 14.5%). However, it is worth noting that the incidence of grade 3 or 4 diarrhea was significantly higher in the N + C group (24.4%) than in the L + C group (12.5%). In view of the severe diarrhea, later the designers of this study firstly reduced the oral dose of capecitabine to 750 mg/m^2^, and secondly added a long-acting bowel motility inhibitor-loperamide to reduce the incidence of diarrhea at the beginning of treatment. Although the incidence of diarrhea remained high, it was generally at a manageable level. Neratinib can cause a certain degree of liver damage that is parallel to associated elevation of liver enzyme levels; in the ExteNET trial(Chan et al. 2016; Deeks 2017), 9.7% of patients had an increased alanine transferase (ALT) level, and elevated aspartate transferase (AST) levels occurred in 5.1% of patients; 1.7% of patients had ALT or AST levels elevated to greater than 5 times the upper limit of normal (ULN), which resulted in discontinuation of medication. In the ExteNET study, the incidence of grade 1–3 diarrhea was significantly higher in the neratinib group than in the placebo group (95% vs. 36%), grade 3 diarrhea occurred in 561 patients (40%) with neratinib, and grade 4 diarrhea occurred 1 patient (1<%) with neratinib, (Chan et al. 2016; Deeks 2017). Notably, antidiarrheal prophylaxis was not specified in the ExteNET study protocol, but diarrhea treatment was instead recommended when associated symptoms became apparent(Dhillon 2019). The open-label, sequential-cohort, phase 2 CONTROL study(Barcenas et al. 2020; Delaloge et al. 2019) (n = 563) was conducted based on the results of the ExteNET study(Chan et al. 2016; Deeks 2017), and it aimed to investigate a preventive regimen for neratinib-associated diarrhea. The results of the CONTROL study(Barcenas et al. 2020; Delaloge et al. 2019) showed that within two weeks of treatment initiation, a dose escalation schedule with either prophylactic antidiarrheal medication or loperamide in addition to neratinib could improve neratinib tolerability and reduce the incidence, severity, and duration of neratinib-related grade 3 diarrhea(Bredin et al. 2020). Together, the main toxicity of neratinib was gastrointestinal side effects which were largely limited to diarrhea, diarrhea was treated with loperamide when symptoms became apparent, and neratinib dose modifications were recommended in cases of grade 2 or 3 diarrhea (in ExteNET).

### Resistance to neratinib

It is estimated that approximately 70% of patients with HER2-positive BC are either innately resistant or have acquired resistance to HER2-targeted drugs(Breslin et al. 2017). Similar to other TKIs, resistance to neratinib may also be generated(Bose and Ma 2021; Segovia-Mendoza et al. 2015). Several possible mechanisms of neratinib resistance have been suggested. Seyhan et al.(Seyhan et al. 2012) screened multiple suppressor genes associated with neratinib resistance using the genome-wide functional RNAi as they described. Neuromedin U (NmU) overexpression was observed in cases with resistance to HER2-targeting drugs, including neratinib(Rani et al. 2014). Adding microRNA-630 (miR-630) to BC cells dramatically enhanced the efficacy of neratinib, and blocking miR-630 induced resistance to neratinib(Corcoran et al. 2014). Zhao et al.(Zhao et al. 2012) found that compared to cells with classical drug resistance, neratinib-resistant cells exhibited downregulation of P-glycoprotein (PGP). Neratinib may inhibit PGP activity and reverse overexpression of PGP. Moreover, neratinib can reverse ATP-binding cassette subfamily B member 1 (ABCB1)-mediated multidrug resistance due to the inhibition of the efflux function of ABCB1(Zhao et al. 2012). CYP3A4 is a cytochrome P450 metabolizing enzyme, and plays a role in the metabolism of approximately half of drugs including neratinib(Breslin et al. 2017). The enhancement of CYP3A4 activity resulted in neratinib resistance in cell line models(Collins et al. 2019). Studies in healthy subjects have shown that co-administration of neratinib and ketoconazole (a strong CYP3A4 inhibitor) increases the plasma concentrations of neratinib(Abbas et al. 2011), and that co-administration of neratinib and lansoprazole (a proton-pump inhibitor, PPI) reduces the plasma concentrations of neratinib(Keyvanjah et al. 2017). Thus, alterations in CYP3A4 activity may also alter the degree of neratinib resistance(Breslin et al. 2017). Hanker et al(Hanker et al. 2017) reported the HER2T798I-mediated neratinib resistance in patients with HER2L869R-mutant BC, and the neratinib resistance could be overcome by other irreversible HER2 inhibitors like afatinib. In addition, the mammalian target of rapamycin (mTOR) pathway alterations which reactivate the HER2 signaling axis and which induce the hyper-activation of the HER kinase signaling are important drivers of neratinib resistance in *HER2* mutant cancers(Collins et al. 2019; Sudhan et al. 2020a, b).

### Neratinib and other TKIs of ERBB2/HER2

Small-molecule anti-HER2 TKIs act on the intracellular domain of the receptor, and mainly include neratinib, lapatinib, pyrotinib, poziotinib and tucatinib which can covalently bind to the intracellular ATP-binding site and inhibit tumor signaling. These TKIs can be divided into two groups, reversible (e.g., lapatinib) and irreversible (e.g., neratinib and pyrotinib) TKIs (Table 2 and Table 3).

**Table 3:** Adverse events summary of TKIs

Adverse events	All Grade	Grade 3/4
	**Neratinib + capecitabine (NALA trial)** **N = 303, n (%)**	
Diarrhea	252 (83.2)	74 (24.4)
Nausea	161 (53.1)	13 (4.3)
PPE syndrome	139 (45.9)	29 (9.6)
Vomiting	138 (45.5)	12 (4.0)
Decreased appetite	107 (35.3)	8 (2.6)
Fatigue	104 (34.3)	9 (3.0)
Constipation	94 (31.0)	4 (1.3)
Stomatitis	62 (20.5)	6 (2.0)
Weight decreased	60 (19.8)	1 (0.3)
Anemia	45 (14.9)	6 (2.0)
Dizziness	43 (14.2)	1 (0.3)
Cough	37 (12.2)	0
Abdominal pain	36 (11.9)	3 (1.0)
Asthenia	36 (11.9)	8 (2.6)
Hypokalemia	35 (11.6)	14 (4.6)
Paronychia	35 (11.6)	2 (0.7)
Pyrexia	33 (10.9)	0
Headache	32 (10.6)	1 (0.3)
Rash	30 (9.9)	0
	**Lapatinib + capecitabine (**Geyer et al. 2006) **N = 164, n (%)**	
Diarrhea	98 (60)	21 (13)
Nausea	72 (44)	3 (2)
Vomiting	43 (26)	3 (2)
Stomatitis	24 (15)	0
Abdominal pain	25 (15)	2(1)
Constipation	16 (10)	0
Dyspepsia	18 (11)	0
Hand–foot syndrome	80 (49)	12 (7)
Rash	45 (27)	2 (1)
Dry skin	18 (11)	0
Fatigue	29 (18)	3 (2)
Mucosal inflammation	18 (11)	0
Asthenia	10 (6)	0
Headache	15 (9)	0
Pain in extremity	21 (13)	1 (< 1)
Back pain	17 (10)	2 (1)
Anorexia	25 (15)	1 (< 1)
Dyspnea	18 (11)	5 (3)
	**Pyrotinib + capecitabine (PHENIX trial)** **N = 185, n (%)**	
Diarrhea	182 (98.4)	57 (30.8)
Hand-foot syndrome	110 (59.5)	29 (15.7)
Nausea	90 (48.6)	0
Vomiting	90 (48.6)	4 (2.2)
White blood cell decreased	84 (45.4)	7 (3.8)
Aspartate aminotransferase increased	71 (38.4)	2 (1.1)
Neutrophil count decreased	68 (36.8)	7 (3.8)
Alanine aminotransferase increased	66 (35.7)	5 (2.7)
Oral mucositis	56 (30.3)	2 (1.1)
Anemia	56 (30.3)	4 (2.2)
Blood bilirubin increased	53 (28.6)	2 (1.1)
Weight loss	48 (25.9)	1 (0.5)
Appetite loss	46 (24.9)	1 (0.5)
Hypokalemia	43 (23.2)	5 (2.7)
Pigmentation disorder	40 (21.6)	0
Bilirubin conjugated increased	35 (18.9)	0
Asthenia	34 (18.4)	1 (0.5)
Hypertriglyceridaemia	27 (14.6)	5 (2.7)
Blood bilirubin unconjugated increased	26 (14.1)	1 (0.5)
Blood creatinine increased	23 (12.4)	0
Platelet count decreased	20 (10.8)	1 (0.5)
	**Tucatinib combination (HER2CLIMB trial)** **N = 404, n (%)**	
Diarrhea	331 (81.9)	53 (13.1)
Nausea	243 (60.1)	16 (4.0)
PPE syndrome	264 (65.3)	57 (14.1)
Vomiting	152 (37.6)	13 (3.2)
Decreased appetite	105 (26.0)	3 (0.7)
Fatigue	193 (47.8)	22 (5.4)
Stomatitis	105 (26.0)	10 (2.5)
Anemia	88 (21.8)	17 (4.2)
Headache	96 (23.8)	3 (0.7)
Aspartate aminotransferase increased	89 (22.0)	19 (4.7)
Alanine aminotransferase increased	85 (21.0)	23 (5.7)
Blood bilirubin increased	81 (20.0)	4 (1.0)
	**Poziotinib (NOV120101-203 trial)** **N = 106, n (%)**	
Diarrhea	102 (96.2)	15 (14.2)
Stomatitis	98 (92.5)	13 (12.3)
Pruritus	67 (63.2)	0
Rash	67 (63.2)	4 (3.8)
Dry skin	41 (38.7)	0
Dermatitis acneiform	34 (32.1)	4 (3.8)
Decreased appetite	32 (30.2)	0
Alopecia	26 (24.5)	0
Nausea	22 (20.8)	0
Mucosal inflammation	21 (19.8)	0
Dyspepsia	16 (15.1)	0
Cough	16 (15.1)	0
Dyspnea	14 (13.2)	2 (1.9)
Vomiting	14 (13.2)	0
Constipation	13 (12.3)	0
Rhinorrhea	13 (12.3)	0
Myalgia	13 (12.3)	0
Fatigue	12 (11.3)	2 (1.9)
Upper respiratory tract infection	12 (11.3)	0
PPE syndrome	11 (10.4)	0
Abdominal pain	11 (10.4)	0

PPE, palmar-plantar erythrodysesthesia. Tucatinib combination: tucatinib, trastuzumab, and capecitabine

The NALA trial(Saura et al. 2020a) suggested that neratinib plus capecitabine significantly improved PFS and time to intervention for CNS disease versus lapatinib plus capecitabine. In the NALA study, higher HER2 expression was associated with a greater benefit for neratinib plus capecitabine compared with lapatinib plus capecitabine(Saura et al. 2021). Compared to lapatinib plus capecitabine, neratinib plus capecitabine was likely to be a cost-effective regimen as third-line therapy for women with HER2-positive MBC (Wu et al. 2022). There data may suggest that neratinib has a better efficacy than lapatinib, with enhanced cost-effectiveness and without increased toxicity.

As an irreversible inhibitor of HER1, HER2, and HER4 originally developed in China, pyrotinib has attracted more and more attention in clinical application due to its many advantages such as easiness to pass through the blood brain barrier and low toxicity(Ma et al. 2019). In 2018, China FDA approved pyrotinib combined with capecitabine to treat HER2-positive advanced/metastatic BC patients who had previously received anthracycline or paclitaxel chemotherapy. In a Phase I study, 38 HER2-positive MBC patients were enrolled, who took 400 mg pyrotinib orally once a day. The most common AE was diarrhea (44.7%), followed by nausea (13.2%), oral ulcer (13.2%), fatigue (10.5%), and leukopenia (10.5%). The most serious AE was grade 3 diarrhea (13.2%Ma et al. 2017b). In a Phase II study to further expand the sample size, 128 patients who had previously used anthracyclines or taxanes (as adjuvant treatment or palliative treatment for relapse and metastasis) were randomly assigned to receive pyrotinib (400 mg once a day) plus capecitabine (1000 mg twice a day) (n = 65) or lapatinib (1250 mg once a day) plus capecitabine (1000 mg twice a day) (n = 63). The ORRs of pyrotinib and lapatinib treatment were 78.5% and 57.1% (*P* = 0.010), respectively. The median PFS was 18.1 and 7.0 months, respectively (*P* < 0.001)(Ma et al. 2019). The PHENIX study was a double-blind, multicenter, randomized phase III study (n = 279) to evaluate the efficacy of pyrotinib plus capecitabine after treatment failure with trastuzumab. The results showed that in patients who failed to paclitaxel and trastuzumab, the combination of pyrotinib and capecitabine could improve PFS compared with capecitabine alone (11.1 versus 4.1 months, *P* < 0.001)(Kunte et al. 2020). The PHOEBE study (n = 267) showed that in MBC patients who previously received trastuzumab, taxus, and/or anthracycline therapy, the PFS for pyrotinib plus capecitabine was better than that for lapatinib plus capecitabine (12.5 versus 6.8 months, *P* < 0.001)(Xu et al. 2021). These suggest that pyrotinib also results in superior survival outcomes compared to lapatinib; however, there have been no direct head-to-head comparisons between the two irreversible HER2-targeting TKIs, neratinib and pyrotinib, so far.

Poziotinib is an irreversible pan-HER TKI that blocks signaling through the HER family of tyrosine-kinase receptors including EGFR, HER2, and HER4(Kim et al. 2019). A total of 75 patients were enrolled in the two phase I studies, the MTDs were determined as 24 mg/day in the intermittent dosing schedule and 18 mg/day in the continuous dosing schedule (Kim et al. 2018). In 51 patients with the intermittent dosing schedule, 8 was PR and 24 was SD, and in 19 patients with the continuous dosing schedule, 4 was PR and 6 was SD. NOV120101-203 trial is a Phase II study to evaluate the efficacy and safety of poziotinib in MBC patients who had failed more than two HER2-directed therapies (Park et al. 2018). The median PFS was 4.04 months, and median OS has not been reached. The main toxicities were diarrhea, stomatitis, and rashes. The incidence of grade 3 or higher AE was 45.3%, and grade 3 diarrhea occurred in 15 patients (14.15%).

Tucatinib is a new oral selective TKI, which is more prone to binding HER2 than HER1 (EGFR)(Murthy et al. 2020).The HER2CLIMB study is a global, double-blind, randomized controlled trial for HER2-positive patients with locally-advanced or MBC (including patients with brain metastasis). Patients were randomly assigned to receive tucatinib or placebo combined with trastuzumab and capecitabine. The median OS was 24.7 months in the tucatinib combination group and 19.2 months in the control group (*P* = 0.004). The median PFS duration was 7.6 months in the tucatinib combination group and 4.9 months in the control group (*P* < 0.001)(Lin et al. 2023). The incidence of grade 3 or higher AE in patients on the tucatinib combination was 60.6%, and grade 3 diarrhea or higher AE occurred in 53 patients (13.1%), grade 3 palmar-plantar erythrodysesthesia (PPE) syndrome occurred in 57 patients (14.1%)(Curigliano et al. 2022). In the population with CNS metastasis, the CNS-PFS (until intracranial progression or death) was 9.9 months in the tucatinib group and 4.2 months in the trastuzumab group, and the median OS was 18.1 and 12.0 months, respectively. The risk of intracranial progression or death in the tucatinib group decreased by 68% (*P* < 0.001)(Lin et al. 2020). Compared with other TKIs, the incidence of diarrhea is low, the dose-limiting toxicity is elevation of transaminase level(Borges et al. 2018). So far there have been no direct comparisons between neratinib and tucatinib.

### Neratinib for HER2-mutant BCs

HER2 mutations are an important oncogenic driver of MBC(Cocco et al. 2018; Floros et al. 2021; Medford et al. 2019). In the phase 1 study by Gandhi et al., 60 patients with HER2-dependent solid tumors (15 BC patients) received neratinib in combination with temsirolimus of different doses. The regimens in the 2 groups were neratinib 200 mg plus temsirolimus 25 mg, and neratinib 160 mg plus temsirolimus 50 mg. Responses were observed in patients with HER2-amplified BC resistant to trastuzumab, with only a minority of patients with HER2-amplified BC showing progressive disease as best response(Gandhi et al. 2014).

The phase 2 SUMMIT trial (NCT01953926) was an open-label, multinational, multicenter basket study to investigate the efficacy and safety of neratinib in solid tumors. In total, 141 patients (125 with HER2-mutant cancers, and 16 with HER3-mutant cancers) received neratinib(Hyman et al. 2018; Oaknin et al. 2020). The trial enrolled 25 HR-positive, HER2-negative MBC patients whose tumors had activating HER2 mutations identified by genome sequencing. Patients were treated with neratinib 240 mg/day orally on a continuous basis with mandatory loperamide prophylaxis. ORR at week 8 as primary endpoint was 32%, and secondary endpoints included CBR of 40% and median PFS of 3.5 months.

Furthermore, Smyth et al. amended the SUMMIT trial by adding a cohort evaluating the combination of neratinib and fulvestrant; they evaluated the efficacy of neratinib, with or without fulvestrant(Smyth et al. 2020), in 81 patients with HER2-mutant MBC, including 34 patients who received neratinib monotherapy (23 were HR-positive and 11 HR-negative) and 47 who received combination therapy (neratinib plus fulvestrant; all were HR-positive). The confirmed ORRs of neratinib monotherapy for ER-positive cancers, neratinib monotherapy for ER-negative cancers, and neratinib plus fulvestrant were 17.4%, 36.4%, and 29.8%, respectively. The rate of prior exposure to cyclin-dependent kinase (CDK)-4/6 inhibitors was higher in the combination therapy cohort than in patients receiving monotherapy (43% versus 12%; *P* = 0.003). The median duration of prior CDK4/6 inhibitor-containing therapy across both cohorts was only 5.0 months. A series of recent cases of HER2 mutations in ER + BC patients following various anti-estrogen therapies have been reported, and in this series, one patient successfully reversed endocrine resistance with the addition of neratinib (Nayar et al. 2019). Interestingly, Smyth et al. have previously shown that at least a subset of these acquired HER2 mutations in HER2-positive BC retain sensitivity to neratinib, despite conferring resistance to HER2-directed monoclonal antibodies and reversible kinase inhibitors(Cocco et al. 2018).

Supporting the results of the SUMMIT trial, the phase 2a clinical plasmaMATCH trial included four parallel cohorts that tested circulating tumor DNA (ctDNA) in 1,000 patients with advanced BC while they were receiving treatment, and examined the relationship between treatment and ctDNA mutations(Turner et al. 2020). Among the cohort B comprised patients with HER2 mutations treated with neratinib, 1 of 20 patients had CR, 4 of 20 patients had PR, and the CBR was 9 (45%) of 20 patients, median PFS was 5.4 months. In the subgroup of patients with HR-positive HER2-negative BC treated with neratinib and fulvestrant, 4 (24%) of 17 patients had a confirmed response. The most common grade 3/4 adverse events were diarrhea (25%, cohort B).

Recently, Shishido et al. used the previously validated high-definition single cell assay (HDSCA) workflow to investigate the clinical significance of liquid biopsy in 5 patients with metastatic BC from the MutHER or SUMMIT trial receiving neratinib and fulvestrant combination therapy. Interestingly, the survival outcomes were associated with the region of HER2 affected. Patients 2, 4, and 5 had a mutation in the intracellular kinase domain, and the best responder Patient 1 had a mutation in the extracellular domain, while the worst responder Patient 3 had a mutation in the transmembrane domain(Shishido et al. 2022).

Based on the results of these studies, neratinib has been proved to have clinical activity on HER2-negative and HER2-mutant BC as well. For HER2-mutant cancers, ORR was 17–36%, which varied according to previous and combined treatment.

### ERBB2/HER2 TKIs for ERBB2/HER2 mutational variants

HER2 mutation is common in and drives the growth of HER2-negative (not HER2-amplified) BC, but it is rare in HER2-positive (HER2-amplified) BC(Cocco et al. 2018). Medford et al. monitored the plasma genotypes in 143 women with endocrine-resistant MBC, and found that patients whose extracorporeal circulating tumor cells (CTCs) had HER2 mutations were highly sensitive to neratinib therapy(Medford et al. 2019).Cocco et al.(Cocco et al. 2018) found that *D769Y* and *D769H* were the most common somatic mutations of HER2; the tumor tissues of patients with *D769Y* mutations were used to establish mouse tumor xenograft models, which were treated with trastuzumab, lapatinib, or neratinib. Trastuzumab and lapatinib were ineffective against the xenograft tumors, while neratinib could effectively inhibit the growth of the tumor(Cocco et al. 2018). Neratinib inhibited BC cells with either wild-type or mutant HER2, and also inhibited the phosphorylation of receptor tyrosine kinase and the HER2 downstream signal protein in these cells(Cocco et al. 2018). In the study by Cocco et al.(Cocco et al. 2018), 6 BC patients with both HER2 amplification and mutation were administered with neratinib; 2 patients had significantly reduced tumor volume, which lasted for 10 months in 1 patient, and 4 patients achieved stabilization of the disease. In the study by Ma et al.(Ma et al. 2017a), 16 patients with HER2-mutated and HER2-nonamplified MBC received neratinib monotherapy, and the CBR was 31%. These data suggested that for certain HER2-mutated BC, neratinib may have a superior efficacy compared to other HER2-targeting TKIs or monoclonal antibodies; however, further clinical investigations and direct comparisons between different drugs are needed.

## Conclusions

### Perspectives and research directions

Due to progress in the treatment of malignant solid tumors, earlier diagnosis by MRI and the aging of the population, the incidence of brain metastases has increased significantly over the past 20 years. The risk of CNS metastases as the site of the first recurrence is low in stage I and II BC patients, but more common in stage III BC patients (Soffietti et al. 2020). With the successful application of HER2-targeted therapy (especially trastuzumab) in the context of metastatic tumors and new diagnoses, the risk and prognosis of CNS in HER2-positive subgroups of patients have changed significantly, resulting in improved control of systemic disease(von Minckwitz et al. 2019). Conversely, trastuzumab is unable to penetrate the intact blood-brain barrier (BBB) at standard doses(Lin et al. 2021) and prevent the occurrence of micrometastases, and there is an increased risk of CNS recurrence. In patients with advanced BC, HER2 overexpression is associated with an increased risk of CNS involvement. The CNS may be a refuge for HER2-overexpressing BC due to its biological peculiarities(Galanti et al. 2021; Shah et al. 2018; Stavrou et al. 2021). Due to the existence of the BBB, the ability of drugs to enter the CNS and exert antitumor function is limited, and the need to improve the clinical prognosis of BCBM is becoming increasingly urgent. Therefore, researches on novel drugs for BCBM are greatly warranted. It is particularly important to develop more effective treatment strategies to manage and ultimately prevent CNS metastasis in BC patients in the future.

Although lapatinib demonstrated that small molecule TKIs of HER2 have the potential to provide some intracranial control, a new generation of neratinib, tucatinib and other emerging HER2 TKIs, have replaced lapatinib in the treatment of HER2-positive BC, especially in patients with multi-line anti-HER2 resistance. Furthermore, the use of the next-generation, pan-HER TKIs in adjuvant setting have the potential to reduce CNS relapse. Many HER2-directed therapies are associated with challenging AE profiles, development of neratinib and the newer pan-HER TKIs has been hampered by gastrointestinal toxicity. For future investigations, it would be interesting to try to expand the treatment indications, e.g., in the neoadjuvant or adjuvant setting, and to further combine hormone therapy with neratinib. Furthermore, we recently found that HER2-targeting antibody-drug conjugates (ADCs) and TKIs may have markedly synergistically-enhanced efficacies when used together, and it would be interesting to explore the safety, feasibility, and efficacy of different combination therapies including neratinib plus anti-HER2 ADCs or other HER2-targeting agents and to identify the optimal combination. Comparisons of neratinib with ADC drugs could help to better clarify the optimal clinical picture of HER2-targeting therapies. In addition to directly targeting the cancer-causing driver HER2 and inhibiting its function, harnessing the innate and acquired immune systems to deal with proliferating cancer cells is a promising and active area of research for HER2-positive BC. Intracranial activity of immune checkpoint inhibitors (ICIs) may be explained by the penetration of the brain through the damaged BBB or meningeal lymphatic vessels, or by the initiation and activation of anti-tumor T cells in the outer parts of the brain and their return to the brain (Swain et al. 2023). However, immunotherapy has not yet shown any significant benefit in HER2-positive BC. Moreover, a number of clinical trials investigating neratinib and other TKIs in novel combination regimens for patients with BC are also ongoing (Table 4). HER2 is also expressed in some other cancers (e.g., gastric cancer), and an investigation of the use of neratinib in such cancers could also be appealing.

**Table 4:** Ongoing clinical trials on TKIs for breast cancer as of March 20, 2023

Registration no.	Status	Cancer type	Study title	Trial phase	No. of subjects	Country
NCT03289039	Active, not recruiting	HER2-positive, ER-positive metastatic breast cancer	A Phase 2 Study of Neratinib With or Without Fulvestrant in HER2-Positive, ER-Positive Metastatic Breast Cancer	Phase 2	21	The US
NCT05388149	Not yet recruiting	HER2-positive breast cancer	Kadcyla And Neratinib for Interception of HER2 + Breast Cancer With Molecular Residual Disease	Phase 2	15	Canada
NCT04965064	Not yet recruiting	HER2-negative metastatic breast cancer	Trial of Neratinib Plus Capecitabine in Subjects With HER2-Negative Metastatic Breast Cancer With Brain Metastases and Abnormally Active HER2 Signaling	Phase 2	22	The US
NCT04366713	Completed	HER2 amplified breast cancer	A Study to Characterize Colon Pathology in Patients With HER2 Amplified Breast Cancer Treated With Neratinib	Phase 2	6	Portugal
NCT04460430	Recruiting	HR-Positive/HER2-negative HER2-enriched advanced/metastatic breast cancer	Targeting EGFR/ERBB2 With Neratinib in Hormone Receptor (HR)-Positive/HER2-negative HER2-enriched Advanced/Metastatic Breast Cancer	Phase 2	56	Multinational
NCT05154396	Not yet recruiting	HER2 positive early breast cancer	Neratinib Dose Escalation Regimen for HER2 Positive Early Breast Cancer	Phase 2	60	China
NCT03377387	Active, not recruiting	Metastatic HER2-positive breast cancer	Capecitabine 7/7 Schedule With Neratinib in Patients With Metastatic HER2-Positive Breast Cancer	Phase 1/2	34	The US
NCT02673398	Active, not recruiting	Stage IV HER2-positive breast cancer	Neratinib in Treating Older Patients With Stage IV HER2-Positive Breast Cancer	Phase 2	25	The US
NCT05252988	Not yet recruiting	Early-stage HER2+, HR + breast cancer	Trial to Evaluate Diarrhoea Discontinuations at 3 Cycles in Patients With Early-stage HER2+, HR + Breast Cancer Treated With Neratinib Plus Loperamide Versus Neratinib Dose Escalation Plus Loperamide Administered as Needed Versus Neratinib Plus Loperamide Plus Colesevelam (DIANER)	Phase 2	315	Spain
NCT05243641	Recruiting	Metastatic breast cancer	Neratinib and Capmatinib Combination (Phase Ib/II) in Metastatic Breast Cancer and Inflammatory Breast Cancer Patients With Abnormal HER2 and c-Met Pathway Activity as Measured by the CELsignia Signaling Analysis Test	Phase 1/2	56	The US
NCT04886531	Not yet recruiting	Triple positive breast cancers	Trial of Pre-operative Neratinib and Endocrine Therapy With Trastuzumab in Triple Positive Breast Cancers	Phase 2	48	The US
NCT04901299	Not yet recruiting	Breast cancer	Fulvestrant + Neratinib In Breast Cancer	Phase 2	25	The US
NCT03812393	Recruiting	Triple negative breast cancer	Evaluating the Efficacy of Neratinib on Live Cell HER2 Signaling Transduction Analysis Positive Triple Negative Breast	Phase 2	27	The US
NCT04388384	Recruiting	Breast cancer	Real-life Pan-HER-blockade With Neratinib (ELEANOR)	Phase 2	200	Germany
NCT03101748	Active, not recruiting	Metastatic or locally advanced breast cancer	Neratinib and Paclitaxel With or Without Pertuzumab and Trastuzumab Before Combination Chemotherapy in Treating Patients With Metastatic or Locally Advanced Breast Cancer	Phase 1/2	43	The US
NCT04760431	Not yet recruiting	HER2 + breast cancer	TKIs vs. Pertuzumab in HER2 + Breast Cancer Patients With Active Brain Metastases (HER2BRAIN)	Phase 2	120	China
NCT05491057	Not yet recruiting	HER2-positive Early-stage Breast Cancer	Treatment Patterns of Neratinib in HER2 + EBC in China	/	500	China
NCT05599334	Recruiting	Early-stage HER2-positive Breast Cancer	A Retrospective Observational Study of Patients With Early-stage HER2-positive Breast Cancer, Treated With Neratinib	/	130	Belgium
NCT03182634	Recruiting	Advanced Breast Cancer	The UK Plasma Based Molecular Profiling of Advanced Breast Cancer to Inform Therapeutic CHoices (plasmaMATCH) Trial	Phase 2	1150	The UK
NCT05760612	Recruiting	Hormone Receptor Positive HER2 -Positive Breast Cancer	A Clinical Study on Hormone Receptor Positive HER2 Positive Breast Cancer of RCB1-2 After Neoadjuvant Treatment With Trastuzumab Combined With Parezumab	Phase 3	300	China
NCT05834764	Recruiting	HER2-positive Breast Cancer	Pyrotinib in Women With High-risk in Early Stage Breast Cancer	Phase 2	188	China
NCT04605575	Recruiting	HER2-positive Breast Cancer	Pyrotinib Plus Vinorelbine in Participants With HER2-positive Previously Treated Locally Advanced or Metastatic Breast Cancer	Phase 2	208	China
NCT05076695	Recruiting	Hormone Receptor Positive HER2 -Positive Breast Cancer	Neoadjuvant With Trastuzumab, Pyrotinib Plus Palbociclib and Fulvestrant in HER2-positive, ER-positive Breast Cancer (NeoTPPF)	Phase 2	37	China
NCT04481932	Recruiting	HER2-positive Breast Cancer	Trastuzumab Combined With Pyrrolidine and Chemotherapy for Locally HER2 Positive Breast Cancer	Phase 2	104	China
NCT05346861	Recruiting	HER2-positive Breast Cancer	Pyrotinib Rechallenge in Her2-positive Metastatic Breast Cancer Pretreated With Pyrotinib and Trastuzumab	Phase 3	240	China
NCT05748834	Recruiting	HER2 + Metastatic Breast Cancer	Study of Tucatinib and Doxil in Participants With Human Epidermal Growth Factor Receptor 2 Positive (HER2+) Metastatic Breast Cancer	Phase 2	36	The US
NCT03975647	Recruiting	HER2-positive Breast Cancer	A Study of Tucatinib vs. Placebo in Combination With Ado-trastuzumab Emtansine (T-DM1) for Patients With Advanced or Metastatic HER2 + Breast Cancer	Phase 3	565	Multinational
NCT05230810	Recruiting	HER2-positive Metastatic Breast Cancer	Clinical Trial of Alpelisb and Tucatinib in Patients With PIK3CA-Mutant HER2 + Metastatic Breast Cancer	Phase 1/2	40	The US
NCT04539938	Recruiting	HER2-Positive Breast Cancer	A Study of Tucatinib Plus Trastuzumab Deruxtecan in HER2 + Breast Cancer (HER2CLIMB-04)	Phase 2	70	The US
NCT04789096	Recruiting	HER2-Positive Breast Cancer	Tucatinib Together With Pembrolizumab and Trastuzumab (TUGETHER)	Phase 2	50	Australia

### Summary

Neratinib is a potent HER2 TKI with high anticancer activity, that it also targets EGFR leading to toxicity. It is not only licensed for adjuvant therapy, but may also play a role in reducing brain recurrence, bringing better treatment option with promising survival benefits to BCBM patients. Neratinib plus capecitabine is superior to lapatinib plus capecitabine, a previous standard therapy prior to the introduction of TDM1. Moreover, the combination of neratinib and fulvestrant also showed the efficacy in HER2 mutations BC. Other TKIs have been developed, the current evidence suggests that tucatinib does not have the same high level of gastrointestinal toxicity due to greater specificity against HER2, but neratinib and tucatinib have never been directly compared. A number of studies with the combination of neratinib are ongoing.

## Data Availability

Not applicable.

## Abbreviations

HER2: Human epidermal growth factor receptor 2
BC: Advanced breast cancer
CNS: Central nervous system
MBC: Metastatic breast cancer
BCBM: Breast cancer brain metastasis
TKIs: Tyrosine kinase inhibitors
FDA: Food and Drug Administration
EGFRs: Epidermal growth factor receptors
pCR: Pathologic complete response
HR: Hormone receptor
GSH: Cys residue of glutathione
ATP: Adenosine triphosphate
ABC: ATP-binding cassette
MAPK: Mitogen-activated protein kinase
FMO: Flavin containing monooxygenase
DLT: Dose-limiting toxicity
MTD: Maximum tolerated dose
NSCLC: Non-small-cell lung cancer
AEs: Adverse events
PR: Partial response
SD: Stable disease
CR: Complete response
TTP: Time-to-tumor progression
T-DM1: Trastuzumab-emtansine
ORR: Objective response rate
PFS: Progression-free survival
CBRs: Clinical benefit rates
DOR: Duration of response
TBCRC: Translational Breast Cancer Research Consortium
iDFS: Invasive disease-free survival
BMs: Brain metastases
WBRT: Whole brain radiotherapy
SRS: Stereotactic radiosurgery
ER: Estrogen receptors
ABCB1: ATP-binding cassette subfamily B member 1
PPI: Proton-pump inhibitor
mTOR: Mammalian target of rapamycin
HDSCA: High-definition single cell assay
CTCs: Circulating tumor cells
BBB: Blood-brain Barrier
BTB: Blood-tumor barrier
CSF: Cerebrospinal fluid
ADCs: Antibody-drug conjugates
ICIs: Immune checkpoint inhibitors
